# Neutrophil Gelatinase-Associated Lipocalin as a Predictor of Acute Kidney Injury in Children With Shock: A Prospective Study

**DOI:** 10.7759/cureus.34407

**Published:** 2023-01-30

**Authors:** Qalab Abbas, Parveen Laghari, Humaira Jurair, Javeria Nafis, Bushra Saeed, Muhammad F Qazi, Ali Saleem, Aysha Habib H Khan, Anwar Haque

**Affiliations:** 1 Department of Pediatrics and Child Health, Aga Khan University Hospital, Karachi, PAK; 2 Department of Pediatrics Pediatric Intensive Care Unit (PICU), The Indus Hospital, Karachi, PAK; 3 Department of Community Health Sciences, Aga Khan University Hospital, Karachi, PAK; 4 Department of Pediatrics and Child Health, Aga Khan University Hospital, Karashi, PAK; 5 Pediatrics, Aga Khan University Hospital, Karachi, PAK; 6 Pathology and Laboratory Medicine, Aga Khan University Hospital, Karachi, PAK; 7 Pediatrics, The Indus Hospital, Karachi, PAK

**Keywords:** shock, serum creatinine, pediatric intensive care unit, neutrophil gelatinase-associated lipocalin, acute kidney injury

## Abstract

Background: The current definition of acute kidney injury (AKI) is based on serum creatinine (SrCr) and urine output, limited by delayed identification of such patients. Plasma neutrophil gelatinase-associated lipocalin (NGAL) is considered an early diagnostic and highly predictive biomarker of AKI.

Objective: To determine the diagnostic accuracy of NGAL for AKI compared with creatinine clearance for early detection of AKI in children with shock receiving inotropic support.

Methods: Critically ill children requiring inotropic support in the pediatric intensive care unit were enrolled prospectively. SrCr and NGAL values were obtained three times at six, 12, and 48 hours after vasopressor initiation. Patients with AKI were defined as having loss of >25% renal function based on creatinine clearance within 48 hours. NGAL level of more than 150 ng/dl was suggestive of the diagnosis of AKI. Receiver operator characteristic curves were generated for NGAL and SrCr to compare the predictive ability of both at 0, 12, and 48 hours of starting vasopressor support.

Results: A total of 94 patients were enrolled. The mean age was 43±50.95 months. Most common primary diagnoses were related to the cardiovascular system (46%). Twenty-nine patients (31%) died during the hospital stay. Thirty-four patients (36%) developed AKI within 48 hours following shock. The area under the curve (AUC) for NGAL at a cutoff of 150 ng/ml was 0.70, 0.74, and 0.73 at six-hour, 12-hour, and 48-hour follow-up, respectively. NGAL had a sensitivity of 85.3% and specificity of 50% at 0 hours of follow-up for diagnosis of AKI.

Conclusion: Serum NGAL has better sensitivity and AUC compared to SrCr for early diagnosis of AKI in children admitted with shock.

## Introduction

Acute kidney injury (AKI) is a complex disorder affecting millions of children worldwide, with a reported incidence of 18-52%, depending on the definition used and the care setting [1-3]. The annual rate of developing AKI in critically ill children is approximately 26.9% [4-6]. Developed countries have reported an annual incidence of 3.3 cases per 100,000 children and a hospital mortality rate of up to 25-50% [6,7]. In developing countries, annual incidence falls between 30-50% [8,9]. Additionally, AKI leads to considerable healthcare utilization, with costs estimated between 8 billion dollars [4]. An early diagnosis is key to preventing morbidity and mortality.

Currently, creatinine clearance (CrCl) is used as a marker of kidney function and is calculated through the modified Schwartz formula [10]. A serum creatinine level (SrCr) is a poor marker for early diagnosis of AKI [11]. It differs with age and gender and fluctuates with hydration, metabolism, and muscle mass [12]. Creatinine does not rise until greater than 50% of renal function is lost. At a lower glomerular filtration rate (GFR) and in acute changes in GFR, the amount of tubular secretion is overestimated. SrCr does not efficiently depict kidney function until steady state equilibrium has been achieved, which may require several days; therefore, it does not reflect accurate kidney function during acute changes in GFR [13]. Some biomarkers with high sensitivity and specificity can be used as an efficient tool for the early detection of AKI, which will help prevent the burden of mortality and morbidity in intensive care units [14-16]. Neutrophil gelatinase-associated lipocalin (NGAL) has recently been identified as a strong marker in predicting early AKI [17,18]. Several published studies have stated that plasma NGAL level rises earlier than SrCr in AKI [19]. Mishra et al.’s study from the US showed that the two-hour sensitivity of NGAL to predict AKI was 70% and specificity was 94% [20]. However, NGAL cutoff values and its performance as an early biomarker of AKI in children with shock is unknown, whereas SrCr is considered a gold standard for diagnosis of AKI as it is a cheaper diagnostic test, although it rises after one to three days of injury. As opposed to SrCr, NGAL is a relatively expensive test (up to 10 times) but a sensitive biomarker in identifying AKI at its early stages. We hypothesize that NGAL rises early in AKI compared to SrCr. Its routine use in patients at risk of AKI may help in earlier than routine (within two hours) institution of reno-protective interventions, which include avoiding nephrotoxic exposures and contrast agents and maintaining euvolemia and perfusion pressure. These measures will not only prevent further renal failure but may also minimize the use of very costly and complicated supportive therapy like continuous renal replacement therapy (CRRT), ultimately resulting in decreased morbidity and mortality related to AKI.

This article was previously presented as a meeting abstract at the Society of Critical Care Medicine Annual Congress on February 5, 2021 (virtual event).

## Materials and methods

Study design and data collection

A prospective study was conducted at the Pediatric Intensive Care Unit (PICU) of Aga Khan University Hospital, an eight-bed multidisciplinary unit with 600 average annual admissions. The study was approved by the ethical review committee of Aga Khan University (ERC #: 4399-PED-ERC-16). Children aged one month to 16 years, admitted to the PICU between May 2016 to April 2018 for more than 48 hours and requiring vasoactive inotropic support, were enrolled prospectively after informed consent from a legal guardian or assent, in case the child was above 12 years. Considering a 95% confidence interval, 80% power, and 10% difference in the area under the curve (AUC) (80% AUC for SrCr and 70% for NGAL) between the two tests in predicting the AKI, 137 children admitted to the PICU were enrolled.

Patients with end-stage renal disease, preexisting renal disease, and those with less than three SrCr levels and serum NGAL samples were excluded. Patients who died before the completion of 48 hours after admission or when baseline SrCr levels were not known were also excluded from the final analysis.

SrCr values and NGAL levels were obtained at three different intervals: 0 hours, 12 hours, and 48 hours. Zero hours was taken at six hours after initiation of an inotropic agent. AKI was diagnosed according to pediatric RIFLE [(risk, injury, failure, loss of function, and end-organ failure) pRIFLE] criteria [21]. pRIFLE was preferred over Kidney Disease Improving Global Outcomes (KDIGO) because it was more commonly used criteria at the time of the study at our center. NGAL level of more than 150 ng/dl was considered suggestive of the diagnosis of AKI. The timing of diagnosis of AKI according to both criteria was at any point in time after the start of an inotropic agent, while a comparison of the two was made up to 48 hours after the start of the inotropic agent. Funding for blood tests was provided by the research council of Aga Khan University.

Laboratory measurements

A total of 1.5ml of blood was collected from an already placed central venous catheter or arterial line. Samples were then sent to the hospital laboratory, where tests were performed for NGAL and creatinine. Serum NGAL was measured quantitatively by a rapid, non-competitive fluorescence immunoassay [19]. The Triage® NGAL test is a point-of-care, fluorescence-based immunoassay used in conjunction with the Triage Meter (Biosite Inc., San Diego, CA, USA) for rapid quantitative measurement of NGAL concentration. The SrCr was measured by Jaffe’s method on ADVIA 1800 Chemistry System (Siemens Healthineers, Erlangen, Germany), and CrCl was calculated by modified Schwartz formula [19].

Data variables were collected on a structured case report form (CRF) which included demographic variables (age, gender, weight, height), clinical and laboratory variables like admission diagnosis, comorbidities, SrCr, and plasma NGAL levels. The discharge status as alive or expired and length of hospitalization were also recorded.

Statistical analysis

Data was entered and analyzed on Statistical Package for Social Sciences (SPSS) version 22 (IBM Corp., Armonk, NY, USA). All normally distributed continuous variables are reported as mean with standard deviation (SD) and are compared between groups by student’s t-test. Non-normally distributed continuous data are reported as medians with interquartile range (IQR) and are compared across groups with the Mann-Whitney U test and the Kruskal Wallis test. All categorical data is reported as proportion and is compared as Fischer’s exact test or Chi-square test. The diagnostic accuracy of plasma NGAL to predict AKI in children with shock is defined in terms of sensitivity, specificity, positive predictive value, and negative predictive value. Receiver operator characteristic (ROC) curve graphs were plotted for NGAL and SrCr to compare the predictive ability of both at 0, 12, and 48 hours of starting vasopressor support. 

## Results

**Figure 1:**
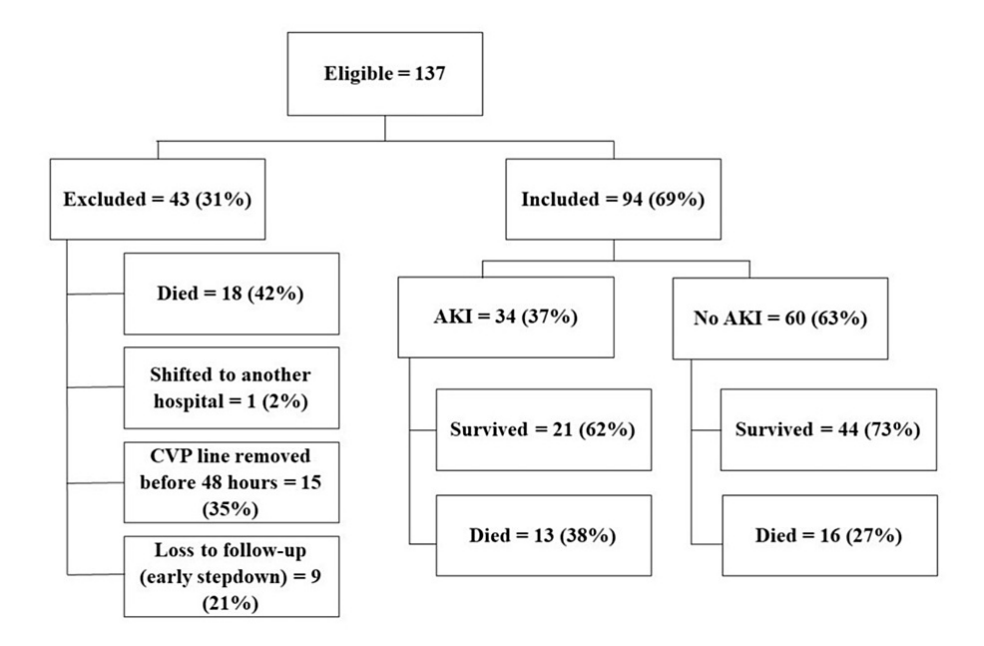
Study Flow Diagram AKI: acute kidney injury, CVP: central venous line, Died: patients expired within 48 hours of admission

A total of 137 eligible patients were prospectively enrolled in the study, of which 18 died in the first 24 hours of hospital admission, nine were lost to follow-up, 15 were excluded due to early removal of the central venous line, and one patient was shifted to another facility. Data collection and follow-up were completed for the remaining 94 patients (Figure 1).

The overall age of the study population was 43.41±50.95 months, and 56 (60%) were males. Major admitting diagnoses were cardiovascular diseases (46%, n=43), septic shock (21%, n=20), central nervous system diseases (12%, n=11), respiratory diseases (10%, n=9), and others (Table 1).

**Table 1:** Demographic and baseline characteristics of the study population CVS: cardiovascular system, CNS: central nervous system, HLOS: hospital length of stay, MODS: multiorgan dysfunction syndrome, AKI: acute kidney injury, SD: standard deviation, IQR: interquartile range, NGAL: neutrophil gelatinase-associated lipocalin, SrCr: serum creatinine *significant: p-value <0.05, a t-test, b chi-square test, c Mann-Whitney U-test

Characteristics	Total n(%)	Non AKI n(%)	AKI n(%)	p-value
	n=94	n=60 (63.83%)	n=34 (36.17%)	-
Age - months (mean ± SD)	43.41±50.95	37.46 ± 43.05	53.91 ±61.84	0.130^a^
Weight - kg (mean ± SD)	12.20±9.43	11.53 ± 8.20	13.40 ±11.32	0.360^ a^
Gender				0.150^b^
Female	38 (40%)	21 (35%)	17 (50%)	-
Male	56 (60%)	39 (65%)	17 (50%)	-
Height - cm (mean ± SD)	86.84±29.29	84.26 ± 25.57	91.38 ± 34.86	0.260^ a^
Diagnosis categories				
CVS	43 (46%)	30 (50%)	13 (38%)	-
Sepsis	20 (21%)	10 (17%)	10 (29%)	-
CNS	11 (12%)	6 (10%)	5 (15%)	-
Respiratory	9 (10%)	8 (13%)	1 (3%)	-
Surgical	7 (7%)	4 (7%)	3 (9%)	-
Abdomen	1 (1%)	1 (2%)	0 (0%)	-
Miscellaneous	3 (3%)	1 (2%)	2 (6%)	-
Mortality				0.240^ b^
No	65 (69%)	44 (73%)	21 (62%)	-
Yes	29 (31%)	16 (27%)	13 (38%)	-
HLOS - Days, (median (IQR))	12 (7-21)	11.5 (7-18.5)	13 (8-28)	0.290^c^
MODS	29 (31%)	12 (20%)	17 (50%)	0.002*
NGAL - ng/ml, (median (IQR))				
at 0 hours	206.5 (107-332)	145 (78-267)	297.5 (200-509)	<0.001^c^*
at 12 hours	199.5 (82-364)	111.5 (67-288.5)	276 (202-555)	<0.001^c^*
at 48 hours	146 (74-323)	110 (71.5-232)	314.5 (150-465)	<0.001^c^*
SrCr - mg/dl, (median (IQR))				
at 0 hours	0.3 (0.2 – 0.5)	0.3 (0.2 – 0.5)	0.3 (0.2 – 0.6)	0.970^c^
at 12 hours	0.4 (0.2 – 0.5)	0.3 (0.2 – 0.45)	0.5 (0.4 – 0.8)	<0.001^c^*
at 48 hours	0.4 (0.3 – 0.6)	0.3 (0.2 – 0.4)	0.8 (0.5 – 1.0)	<0.001^c^*

Ninety-four patients were divided into two groups, those who developed AKI (AKI group; 37%, n=34), and those who did not (non-AKI group; 64%, n=60). Both groups did not differ significantly in characteristics like age, weight, height, and diagnosis (Table 1). Thirty-eight percent (13/34) of patients in the AKI and 27% (16/60) in the non-AKI group expired (p=0.24). The median length of hospital stay was 11.5 days (seven to 18.5) and 13 days (eight to 28) for non-AKI and the AKI groups, respectively (p=0.29). Multiorgan dysfunction syndrome (MODS) developed in 17 (50%) patients in the AKI group and 12 (20%) patients in the non-AKI group (p=0.002) (Table 1).

Median serum NGAL levels at 0, 12, and 48 hours for the AKI group were 297 ng/ml (200-509), 276 ng/ml (202-555), and 314 ng/ml (150-465), respectively. Median serum NGAL levels for the non-AKI group at these intervals were 145 ng/ml (78-267), 111.5 ng/ml (67-288.5), and 110 ng/ml (71.5-232), respectively, (p<0.001). Median serum creatinine values at 0 hours were 0.3 (0.2-0.5) and 0.3 (0.2-0.6) for the non-AKI and AKI groups, respectively (p=0.970). At 12 and 48 hours after the start of inotropic support, serum creatinine was 0.5 (0.4-0.8) and 0.8 (0.5-1.0) for the AKI group (p<0.001) (Table 1).

At a cutoff of NGAL value of 150 ng/ml, sensitivity and specificity for AKI diagnosis keeping pRIFLE at 12 hours as the gold standard was 85.3% (68.9-95), 88.2% (72.5-96.7) and 76.5% (62.1-91.3) at 0, 12 and 48 hours (Table 2). While comparing pRIFLE and NGAL for AKI, NGAL diagnosed 29 and 20 more patients than pRIFLE at 12 and 48 hours (Table 3).

**Table 2:** Sensitivity, specificity, and positive and negative predictive value of neutrophil gelatinase-associated lipocalin in predicting acute kidney injury at a cutoff level of 150 ng/ml at three time points PPV: positive predictive value, NPV: negative predictive value, CI: confidence interval

Time point	Sensitivity (95% CI)	Specificity (95% CI)	PPV	NPV
0 Hours	85.3% (68.9,95)	51.7.0% (36.8,63.2)	49.20%	85.70%
12 Hours	88.2% (72.5,96.7)	56.7% (43.2,69.4)	53.60%	89.50%
48 Hours	76.5% (62.1,91.3)	66.7% (53.3,78.3)	57.40%	85.10%

**Table 3:** Acute kidney injury as diagnosed by pRIFLE criteria and neutrophil gelatinase-associated lipocalin (cutoff 150 ng/ml) in 94 patients AKI: acute kidney injury, NGAL: neutrophil gelatinase-associated lipocalin, pRIFLE: pediatric risk, injury, failure, loss and end-stage renal disease criteria

AKI as diagnosed by NGAL	AKI as diagnosed by pRIFLE			
	12 hours		48 hours	
	Yes	No	Yes	No
No	30	8	40	7
Yes	29	27	20	27

**Figure 2:**
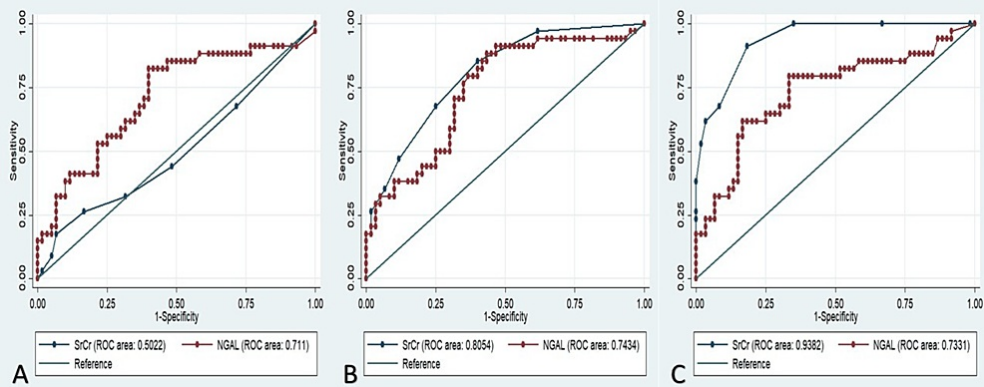
Comparison of serum creatinine and neutrophil gelatinase-associated lipocalin for diagnosis of acute kidney injury (ROC curve) at 0 hours (A), 12 hours (B) and 48 hours (C) SrCr: serum creatinine, NGAL: neutrophil gelatinase-associated lipocalin, ROC: receiver operating curve

At 0 hours, the diagnostic accuracy of NGAL for predicting AKI was 70% (AUC 0.7, CI-0.59,0.82), whereas that of serum creatinine was 50% (AUC 0.5, CI-0.37,0.6). At 12-hour follow-up, NGAL and serum creatinine have almost similar diagnostic accuracies, whereas at 48 hours serum creatinine with an AUC of 0.94 (CI-0.89,0.98) supersedes the predictive ability of NGAL (AUC 0.73, CI-0.62,0.84) (Figure 2). Median NGAL values at three points were different between both groups (p<0.001), however, at three points were the same or increased in the AKI group and decreased serially in the non-AKI group (Table 4).

**Table 4:** Comparison of neutrophil gelatinase-associated lipocalin values between AKI and non-AKI groups AKI: acute kidney injury, NGAL: neutrophil gelatinase-associated lipocalin ϯ median. Mann-Whitney U-test: ¹p-value=0.0008, ²0.0002 *Friedman Test: p-values in columns, for differences between NGAL values taken at different timepoints within each group NA not applicable

NGAL^ϯ ^(ng/ml)	AKI group				Non-AKI group			
	T1	T2	T3	p-value	T1	T2	T3	p-value
	297.5^NA^	276¹	314.5²	<0.001*	145^NA^	111.5¹	110²	<0.001*

## Discussion

We showed that NGAL has better sensitivity and negative predictive value for early diagnosis of AKI in children with various underlying diagnoses receiving vasopressor support, compared to SrCr, NGAL diagnosed more children with AKI at 12 and 48 hours compared to pRIFLE (creatinine). Another key finding from our study is that the NGAL value remained elevated in children who developed AKI compared to those who did not; the same has also been shown previously by Wheeler et al. [22]. We used time zero as the six hours after the start of inotropic support, assuming that it is a more serious condition compared to a shock reversed by only fluid therapy; because we wanted to test the utility of NGAL as an early predictor of AKI.

AKI is a complex problem in children with a spectrum ranging from mild asymptomatic dysfunction to complete renal shutdown and anuria [23]. NGAL is strongly upregulated among damaged renal tubular epithelial cells, which start rising 48 hours before SrCr and provide early diagnosis of AKI [23]. Shock (especially septic and cardiogenic) is a common reason for admission to PICU and also a leading precipitating cause of AKI [1]. Our study showed a highly significant difference between serum NGAL in patients with shock who had AKI and who did not, similar to that shown by Afify et al. and Wheeler et al. in their studies of children with sepsis [22,24]. We included patients who had fluid refractory shock and required inotropes for at least six hours, because investigators had observed that many patients in our PICU receive inotropes for less than six hours, which may be due to reasons other than shock (for example optimization of cardiac output in coma etc).

At the cutoff value of serum NGAL of 150 ng/ml, the sensitivity for diagnosing AKI was 85%, slightly lower than the 89% sensitivity reported for adults (247 ng/ml) and 70% specificity for AKI prediction. This is like another study in children with septic shock, which showed an AUC of 0.67 at a cutoff value of 139 ng/ml with similar sensitivity and specificity [22,25]. At 48 hours, creatinine performed better than NGAL. This is against the previous evidence and could be because ongoing damage had stopped, and creatinine kept rising as it rises late. Note that the overall cohort was critically ill, as shown by the fact that 18 patients died within the first 24 hours, another 29 (31%) died during the hospital stay, and a similar number had MODS [24].

Moreover, most of our patients had an underlying cardiac diagnosis (almost all of these had open heart surgery for congenital heart disease), where NGAL has been shown to be an early predictor of AKI [26]. The second most common diagnosis was sepsis with or without septic shock, a diagnosis where NGAL has an excellent predictive ability for AKI in children [22]. Patients who developed AKI had more MODS than those who did not. NGAL has been proposed to be a marker of the development of MODS; however, we could not study this [22]. Our study indicated no statistically significant difference in mortality between those with AKI and those without AKI. This is probably because our study was not powered to detect this difference.

NGAL remains one of the early biomarkers of AKI in critically ill children. Recently there have been other alternatives for early diagnosis of AKI, like urinary NGAL, renal angina index, precision medicine approach using biomarkers and a combination of cell damage biomarkers and functional biomarkers [27-29]. Our study provides evidence that serum NGAL can be used as an early predictor of AKI in a diverse population of children admitted to the PICU at risk of developing AKI.

However, our results should be interpreted keeping in mind some limitations. Serum NGAL as a predictor of AKI or for the diagnosis of AKI has its limitation since it is released from multiple sites in the body and can be affected by other underlying conditions like sepsis, urinary NGAL may be a good alternative. We used SrCr exclusively without urinary criteria for AKI diagnosis. Hence, we may have underestimated the incidence of AKI. Kellum et al. showed that AKI could occur without affecting urine output [30]. Certain interventions, like diuretics, mannitol, and dopamine, can also affect urine output. Results might also be affected by a significant number of excluded participants for reasons mentioned in Figure 1. Due to the limited sample size, we could not perform a sub-group analysis. A large multicenter study is needed to confirm these findings.

## Conclusions

In conclusion, serum NGAL has proven to be a sensitive biomarker for early predicting AKI in critically ill children with shock receiving vasopressor compared to SrCr levels. It also effectively diagnosed more children with AKI compared to pRIFLE. Hence, routine use of NGAL in those at risk of AKI can aid in early diagnosis and starting timely preventive and management strategies can help alter the usual course of disease which can result in better outcomes in children with AKI.
